# Global research trends and hotspots in the pathophysiology, imaging and therapy of type 3 macular neovascularization (MNV3) in age-related macular degeneration (AMD)

**DOI:** 10.3389/fmed.2026.1811978

**Published:** 2026-04-24

**Authors:** Ye Li, Xiangyu Fu, Ling Huang, Lirong Xiao, Danian Chen

**Affiliations:** 1 Department of Ophthalmology, West China Hospital, Sichuan University, Chengdu, China; 2 Research Laboratory of Ophthalmology and Vision Sciences, Eye Research Institute, West China Hospital, Sichuan University, Chengdu, China

**Keywords:** age-related macular degeneration, type 3 macular neovascularization, VEGF, optical coherence tomography, CiteSpace, VOSviewer, bibliometric analysis

## Abstract

**Background:**

To provide an overview of the research hotspots and directions of type 3 macular neovascularization (MNV3) in age-related macular degeneration by bibliometric analysis.

**Methods:**

Publications related to MNV3 were retrieved from two databases, the Science Citation Index-Expanded database of the Web of Science Core Collection (WoSCC) and PubMed. CiteSpace was utilized to analyze country distribution, keyword bursts, and co-cited references. VOSviewer was employed to identify collaborative authors and the keyword co-occurrence network.

**Results:**

A total of 564 publications were identified from 2001 to 2026. They were performed in 97 countries, with the United States leading the way. Kim JW is the most prolific author, while Yannuzzi LA is the most cited author. *Investigative Ophthalmology & Visual Science* has published the most papers. *Retina-The Journal of Retinal and Vitreous Diseases* is the most cited journal. In the keyword co-occurrence network, apart from terms related to MNV3, “ranibizumab,” “photodynamic therapy” and “optical coherence tomography” are high-frequency keywords, while terms such as “artificial intelligence,” “biomarker,” and “microglia” have emerged as more recent research hotspots. Research topics can be categorized into four major clusters: pathogenesis, imaging, therapy, clinical prognosis and long-term management. Meanwhile, highly cited co-cited articles successively addressed risk stratification, early identification, imaging evaluation, and standardized therapeutic regimens of MNV3, providing important references for clinical practice.

**Conclusion:**

This bibliometric analysis elucidates research hotspots and directions in MNV3 across pathophysiology, imaging, and therapy, and outlines the dynamic development of research in this field.

## 1 Introduction

Age-related macular degeneration (AMD) is a chronic, progressive retinal disease characterized by extracellular deposit accumulation and photoreceptor degeneration under multifactorial impact. As the most common cause of blindness in developed countries (1, 2), AMD can be classified into dry and wet forms. Dry AMD (85–90% of cases) manifests as dysfunction of the retinal pigment epithelium (RPE) and photoreceptor loss. In contrast, wet AMD (10–15% of cases), also known as neovascular AMD (nAMD), presents as macular neovascularization (MNV) and is responsible for 90% of severe vision loss affected by AMD (3, 4). Based on imaging findings, wet AMD is subdivided into three types: MNV1, MNV2, and MNV3. MNV1 corresponds to the historical term “occult choroidal neovascularization (CNV).” MNV2 represents “typical CNV.” MNV3, previously termed “retinal angiomatous proliferation (RAP),” is a retinal-derived neovascular lesion originating from the retinal deep capillary plexus, penetrating the RPE layer, and anastomosing with choroidal vessels (5). Recent studies on MNV3 have indicated that the incidence of MNV3, as detected by optical coherence tomography (OCT), appears to be much higher than previously recognized (6–8). It is strongly associated with reticular pseudodrusen (RPD), with predictable progression to macular atrophy (MA) and worse visual prognosis (9). Furthermore, while anti-vascular endothelial growth factor (anti-VEGF) therapy remains the first-line treatment, its clinical efficacy is limited by disease heterogeneity, and it can be associated with serious complications like MA (9).

Recently, with in-depth research on MNV3, there is significant development potential in this field, as multi-targeted therapeutic options have been gradually advanced and artificial intelligence (AI) has assisted in more accurate diagnosis of MNV3 (10–12). Although interest in MNV3 is growing, a dedicated bibliometric study of this subfield is lacking. This gap has left the global research landscape, core collaborative networks, and the evolution of research topics unclear. Bibliometrics offers quantitative methods for reviewing and analyzing existing literature in specific fields through statistical analysis of publications (13). The integration of visualization analysis further enhances the value of bibliometric studies in medical research, with applications already established across multiple ophthalmology subspecialties (14, 15). Therefore, this study applies bibliometric and visual analysis to map the research hotspots, intellectual structure, and emerging directions in MNV3 research, thereby providing a foundation for future investigations in this evolving field.

## 2 Methods

### 2.1 Data sources and search strategies

**FIGURE 1:**
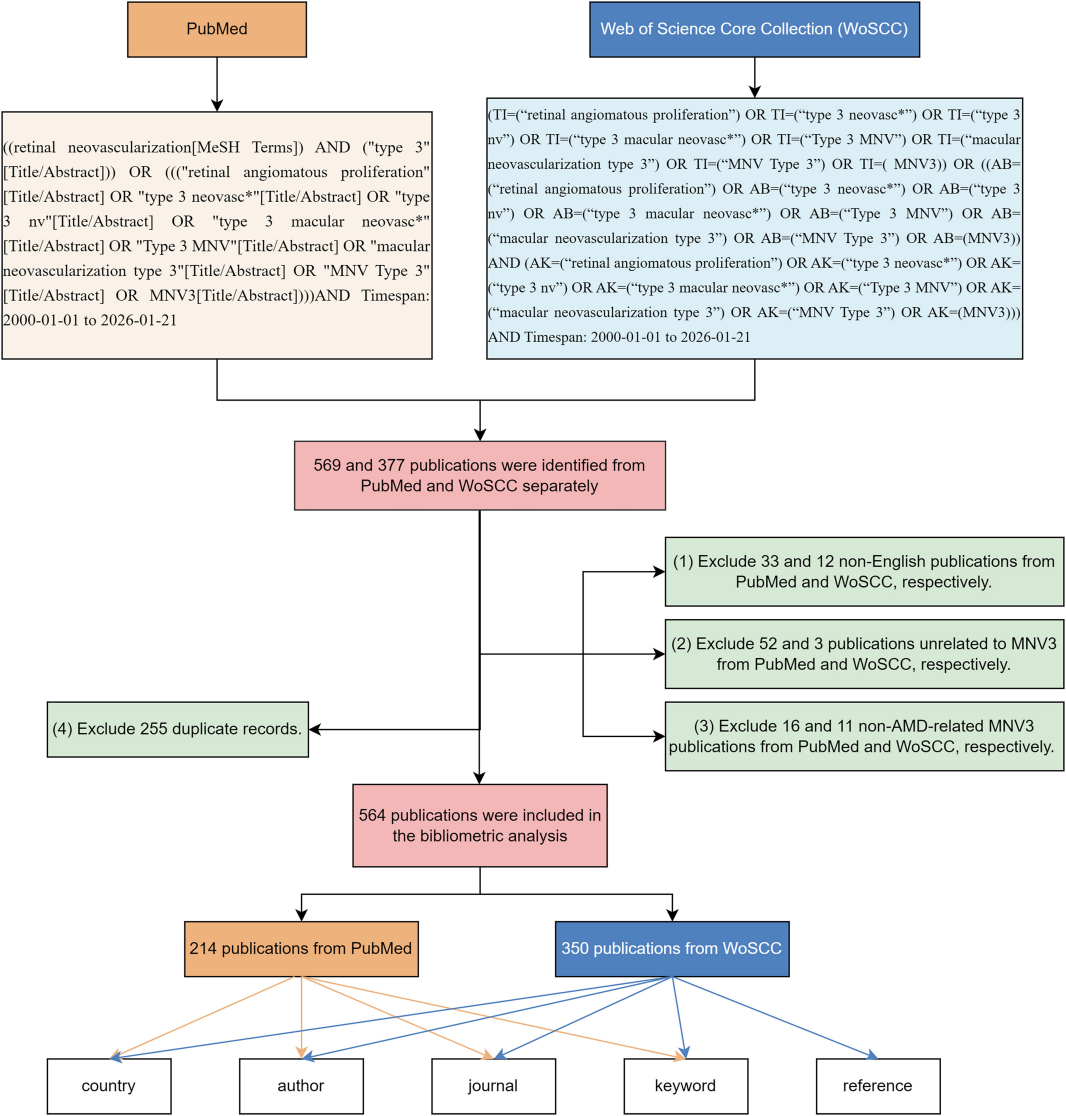
The flow chart of the searching, screening and analysis.

We identified the Science Citation Index-Expanded (SCI-E) of Web of Science Core Collection (WoSCC) and PubMed for all MNV3-related literature on January 21, 2026. To ensure the completeness and accuracy of the search, we employed a comprehensive set of search terms encompassing all variants associated with the key concepts of “retinal angiomatous proliferation,” “type 3 neovascularization,” and “type 3 macular neovascularization,” covering the period from January 1, 2000, to January 21, 2026. A total of 377 and 569 publications were initially retrieved from WoSCC and PubMed, respectively. We first excluded non-English literature. Then, by reading all the titles and abstracts, and skimming through the full texts of articles with vague expressions, 564 publications (350 from WoSCC and 214 from PubMed, excluding 255 duplications) were finally included according to the following exclusion criteria: (1) unrelated to MNV3; (2) not AMD-related MNV3 (Figure 1). All information on eligible publications was exported and saved in plain-text format, including journal sources, titles, authors, research institutions, abstracts, keywords and references.

### 2.2 Data reliability

To ensure data accuracy and reliability for the bibliometric analysis, rigorous quality control procedures were applied throughout the literature retrieval, screening, extraction, and deduplication stages. (1) Retrieval and screening were completed, followed by predefined inclusion and exclusion criteria. (2) In the deduplication phase, R was first used to remove duplicates based on DOI; entries without a DOI were deduplicated by matching title, authors, and year. After algorithmic deduplication, manual review was conducted to further remove duplicate entries and ensure that all included publications were independent and valid. (3) Detailed records were maintained for each step, encompassing the search strategies, excluded articles (with counts and titles), and duplicate records (with counts and entries), thereby guaranteeing full traceability and transparency throughout the study.

### 2.3 Bibliometric analysis

For the analysis of country and author collaboration networks and keyword co-occurrence, we adopted a merged dataset from both WoSCC and PubMed databases. Given the inherent limitations of PubMed-retrieved data in citation analysis, all citation-related analyses (e.g., reference clusters) were conducted exclusively with the WoSCC database to complement and validate the research trends.

CiteSpace V6.4.R1 and VOSviewer V1.6.20 are essential tools for visualization analysis (16, 17). CiteSpace was applied to analyze and visualize the distribution of countries/regions, keyword citation bursts, and co-cited references. VOSviewer was employed to create co-occurrence networks of collaborating authors and keywords.

CiteSpace parameters included annual time slicing (2001–2026), node types (“country” and “reference”), Pathfinder pruning, and default settings otherwise. Significant burst keywords were defined as at least a twofold increase in frequency and a minimum duration of two years. The cluster labels were generated from the title field of the citing documents within each partition using the Log-Likelihood Ratio (LLR).

Clustering quality was evaluated using the silhouette (S) and modularity (Q). The S score measures intra-community cohesion and inter-community separation from the perspective of individual nodes (18), whereas the Q score assesses the overall strength of the community structure (19). The closer the S and Q values are to 1, the better the clustering performance. Typically, communities with S > 0.5 and Q > 0.3 are considered robust (18, 20).

## 3 Results

### 3.1 Analysis of the number of publications

**FIGURE 2:**
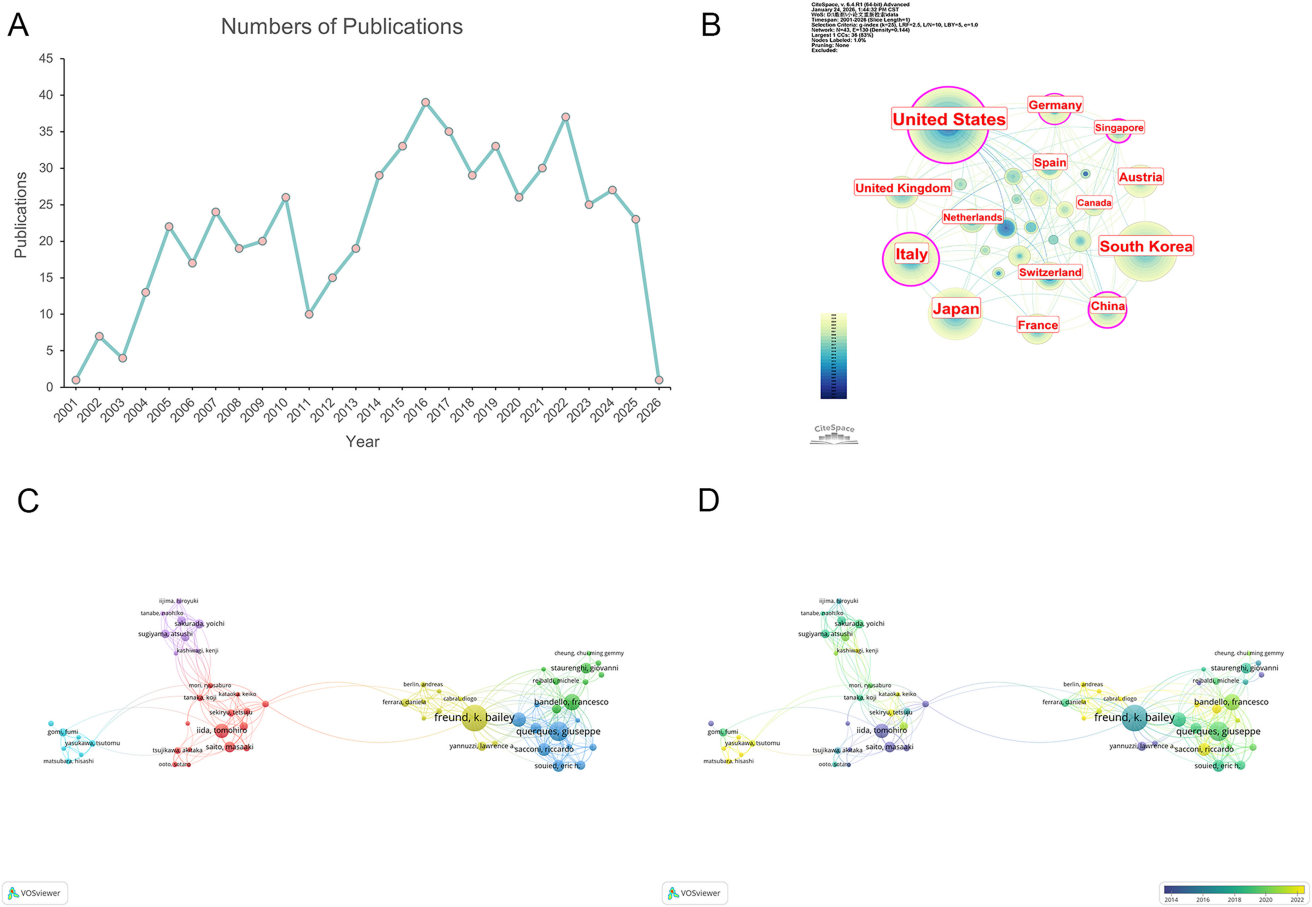
Contributions and collaboration by year, country, and author. **(A)** Trends in the annual number of MNV3-related publications. **(B)** Country distributions of the publications. The node size corresponds to the number of publications. Purple rings on the periphery mean a high centrality. **(C)** Visualization of author collaboration. **(D)** Colour mapping by the average year of author collaboration.

The number of publications can reflect the research progress in a field, and Figure 2A illustrates the publication trend of MNV3 from 2001 to 2026. It demonstrates a dynamic evolutionary trajectory with distinct phases. The initial period (2001–2010) exhibited a fluctuating yet overall upward trend in publications, followed by a decline around 2011. Subsequently, publication numbers rebounded strongly, eventually exceeding the earlier growth levels. In general, research in this field is continuously progressing.

### 3.2 Analysis of leading countries

A total of 97 countries/regions contributed to MNV3-related research. Table 1 lists the top eight countries by publication counts. The United States ranked first (142, 25.18%), followed by Japan (90, 15.96%) and Italy (89, 15.78%). The country collaboration network (Figure 2B) revealed high centrality for the United States, China, Italy, and Germany. Singapore also exhibited high centrality (0.25) despite its low publication output. Remarkably, although the United Kingdom ranked only eighth in publications, it far exceeds fifth-place Austria in citation counts, underscoring its distinctive academic influence.

**TABLE 1:** Publications in the eight most productive countries/regions.

Rank	County	Centrality	Counts (%)	Citation
1	United States	0.37	142(25.18)	3289
2	Japan	0.09	90(15.96)	757
3	Italy	0.25	89(15.78)	1034
4	South Korea	0.00	77(13.65)	749
5	Austria	0.00	30(5.32)	180
6	China	0.30	29(5.14)	344
7	Germany	0.12	29(5.14)	145
8	United Kingdom	0.08	29(5.14)	446

### 3.3 Analysis of authors and co-cited authors

A total of 2089 authors were involved in MNV3-related research. Table 2 presents the top 10 productive and co-cited authors in WoSCC. As shown in Table 2, half of the top 10 most prolific authors are from Konyang University, South Korea, while six of the top 10 co-cited authors are from the United States. Hartnett ME from Stanford University was the first researcher to report this disease (21, 22). Subsequently, a highly collaborative team from the Manhattan Eye Ear and Throat Hospital, comprising Yannuzzi LA, Freund KB, Spaide RF and Gross NE, has made substantial contributions through their joint publications.

**TABLE 2:** Top 10 authors and co-cited authors.

Rank	Authors	Counts	County	Co-cited authors	County	Citation counts
1	Kim JW	31	South Korea	Yannuzzi LA	United States	307
2	Kim JH	29	South Korea	Freund KB	United States	220
3	Freund KB	26	United States	Kim JH	South Korea	213
4	Kim CG	22	South Korea	Spaide RF	United States	150
5	Querques G	19	France	Hartnett ME	United States	124
6	Lee DW	15	South Korea	Querques G	France	103
7	Bandello F	14	Italy	Gass JDM	United States	100
8	Sarraf D	14	United States	Boscia F	Italy	96
9	Schmidt-Erfurth U	14	Austria	Bottoni F	Italy	87
10	Chang YS	12	South Korea	Gross NE	United States	75

Figure 2C illustrates the largest collaborative network among authors with more than 5 documents, divided into 6 clusters. Notably, temporal analysis clearly demonstrates that researchers led by Freund KB, Querques G, Bandello F and Iida T have conducted sustained in-depth research in this field (Figure 2D). Freund KB, Querques G, Bandello F and their colleagues focus on imaging-related pathophysiological changes and biomarkers of MNV3, while Iida T’s team explores the therapeutic efficacy, prognostic factors and associated economic burden of MNV3.

### 3.4 Analysis of journals and co-cited journals

All papers have been published in 86 journals. Table 3 lists the top 10 productive journals and co-cited journals in WoSCC, with *Investigative Ophthalmology & Visual Science* (*IOVS*) hosting the highest number (77, 22%), followed by *Retina - The Journal of Retinal and Vitreous Diseases* (*Retina*) (75, 21.43%) and *Graefe’s Archive for Clinical and Experimental Ophthalmology* (36, 10.29%). The top 10 journals by co-citation frequency highly overlap with the top 10 by publication volume. *Retina* topped the list of co-cited journals with a substantial citation count of 2019, indicating its significance in disseminating influential studies. *Ophthalmology* ranked second with 814 citations, followed by *American Journal of Ophthalmology* (783) and *Graefe’s Archive for Clinical and Experimental Ophthalmology* (525).

**TABLE 3:** Top 10 journals and co-cited journals.

Rank	Journal	Counts (%)	JCR (2024)	Co-cited journal	Citation counts	JCR (2024)
1	Investigative Ophthalmology and Visual Science	77(22.00)	Q1	Retina - The Journal of Retinal and Vitreous Diseases	2019	Q2
2	Retina - The Journal of Retinal and Vitreous Diseases	75(21.43)	Q2	Ophthalmology	814	Q1
3	Graefe’s Archive for Clinical and Experimental Ophthalmology	36(10.29)	Q2	American Journal of Ophthalmology	783	Q1
4	American Journal of Ophthalmology	15(4.29)	Q1	Graefe’s Archive for Clinical and Experimental Ophthalmology	525	Q2
5	Acta Ophthalmologica	14(4.00)	Q1	Archives of Ophthalmology	465	Q1
6	Ophthalmologica	14(4.00)	Q3	British Journal of Ophthalmology	403	Q1
7	British Journal of Ophthalmology	13(3.71)	Q1	Investigative Ophthalmology & Visual Science	345	Q1
8	European Journal of Ophthalmology	12(3.43)	Q3	Eye	153	Q1
9	Eye	12(3.43)	Q1	Acta Ophthalmologica	101	Q1
10	Japanese journal of ophthalmology	7(2.00)	Q2	European Journal of Ophthalmology	98	Q3

### 3.5 Analysis of co-occurring keywords and burst terms

The analysis of keywords can reflect the research hotspots in the field. In this study, synonyms, singular/plural variations, and different spellings were initially merged. Table 4 summarizes the top 25 keywords, which fall into three categories: pathology and subtypes of MNV3 (e.g., “retinal angiomatous proliferation”), diagnostic techniques (e.g., “optical coherence tomography”), and therapeutic strategies (e.g., “ranibizumab”).

**TABLE 4:** Top 25 keywords.

Rank	Keywords	Counts
1	Age-related macular degeneration	245
2	Retinal angiomatous proliferation	220
3	Choroidal neovascularization	109
4	Ranibizumab	93
5	Photodynamic therapy	90
6	Optical coherence tomography	86
7	Type 3 neovascularization	75
8	Vascular endothelial growth factor	61
9	Bevacizumab	60
10	Anti-vegf therapy	51
11	Triamcinolone	49
12	Neovascularization	46
13	Anastomosis	45
14	Geographic atrophy	43
15	Polypoidal choroidal vasculopathy	39
16	Optical coherence tomography angiography	38
17	Aflibercept	36
18	Type 3 macular neovascularization	36
19	Occult choroidal neovascularization	36
20	Verteporfin	32
21	Neovascular age-related macular degeneration	31
22	Retinal pigment epithelium detachment	31
23	Indocyanine green angiography	30
24	Reticular pseudodrusen	30
25	Drusen	28

**FIGURE 3:**
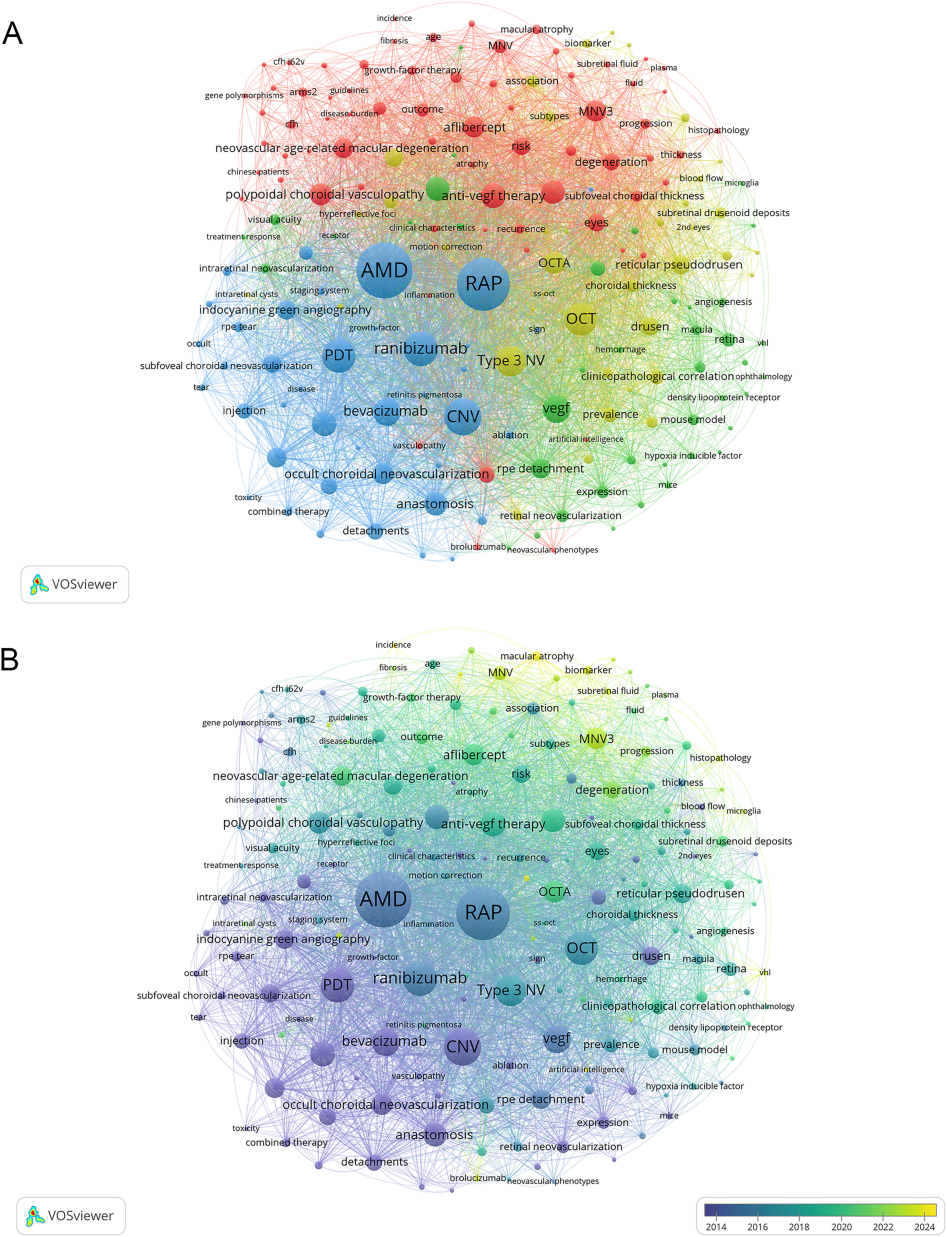
The mapping of keywords about MNV3. **(A)** Keyword co-occurrence network. The size of the node represents the frequency of occurrences. **(B)** Colour mapping by the average year of keyword occurrence. Keywords in yellow appeared later than those in blue.

Figure 3A shows the keyword co-occurrence network generated by VOSviewer, with keywords that appeared at least twice intuitively classified into four clusters. MNV3 pathogenesis and basic research form the core of the green subset with keywords such as “VEGF expression,” “density lipoprotein receptor,” and “mouse model.” Imaging-based diagnosis and assessment are highlighted in the yellow group, which includes “OCT,” “OCTA,” and “reticular pseudodrusen.” The blue partition is dedicated to therapeutic approaches, involving “PDT,” “ranibizumab,” and “bevacizumab.” The red zone emphasizes clinical research and precision medicine, with studies in specific populations (e.g., “Japanese patients,” “Chinese patients”), treatment regimens (e.g., “treat-and-extend regimen,” “pro re nata regimen”), as well as disease prevalence, recurrence patterns and so on.

As shown in Figure 3B, the research focus has shifted from conventional drug therapies and procedural interventions toward precision medicine, with the aim of optimizing treatment schedules and outcome measures. A key example is the comparison between treat-and-extend (TAE) regimens and *pro re nata* (PRN) regimens to refine dosing schedules (23, 24). Concurrent advances in pathology are enhancing the understanding of MNV3. For instance, investigators are exploring diverse biomarkers, such as intraretinal hemorrhage (IRH), intraretinal fluid (IRF), hyperreflective foci (HRF), retinal thickness (RT), and subretinal fluid (SRF), and employing AI models to quantify these markers for diagnostic assistance (25–27). Interestingly, the first four biomarkers mentioned are considered biomarkers for MNV3. For SRF, a novel quantitative AI showed it was a biomarker of choroidal origin in MNV1 (27). At the same time, other research has found that its presence in MNV3 may indicate more advanced disease with a high risk of visual deterioration (28). Furthermore, the mechanism of neuroinflammation involving microglia has emerged as an area of active research in the pathogenesis of MNV3. A growing body of evidence indicates that activated microglia accumulate in MNV3 lesions and drive pro-inflammatory responses (29). Key microglia-related molecules and pathways, such as secreted phosphoprotein 1 (SPP1) and receptor-interacting protein kinase 3 (RIP3)-dependent necroptosis, may serve as potential therapeutic targets, and targeted inhibition may complement existing anti-VEGF treatment strategies (30, 31).

**FIGURE 4:**
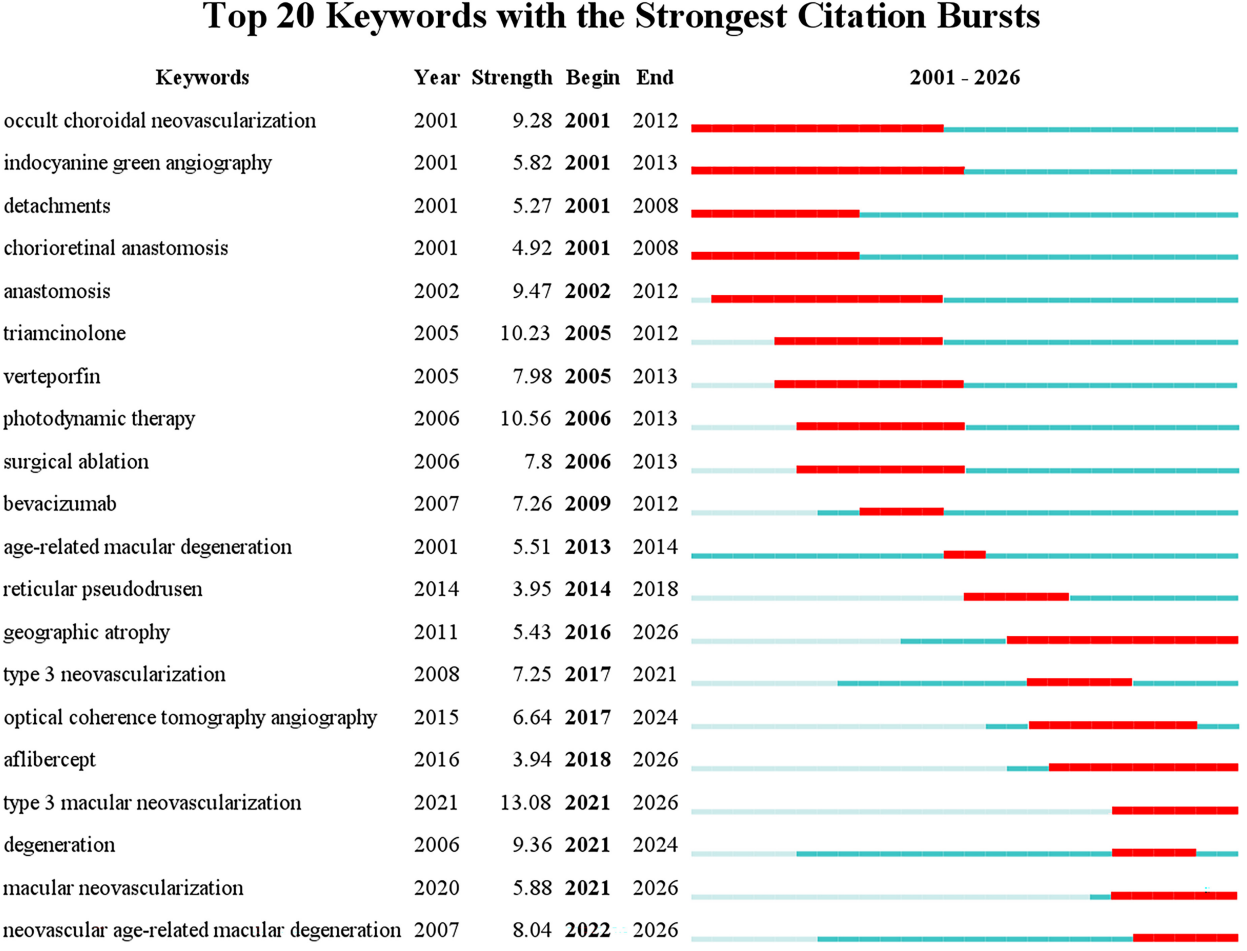
The top keywords with the strongest citation bursts in MNV3-related research.

Beyond the keyword co-occurrence network, the keyword bursts analysis can show research hotspots at specific times. The top 20 keywords with the strongest citation bursts (Figure 4) clearly delineate two distinct developmental phases.

The early phase (2001–2014) featured terms like “occult choroidal neovascularization” and “chorioretinal anastomosis,” reflecting a period when MNV3 was not yet clearly distinguished from CNV. This diagnostic ambiguity stemmed from their clinical and pathological overlap, in which the chorioretinal anastomosis in MNV3 was hard to differentiate from CNV vascular abnormalities (32). Furthermore, conventional fluorescein angiography could not trace the origin of abnormal vessels (33). Although the distinct pathogenesis of MNV3 was not fully elucidated during this period, its association with inflammation and VEGF-driven proliferation had been recognized. Therefore, various treatment approaches were explored, including anti-inflammatory therapy, anti-VEGF therapy, photodynamic therapy (PDT), and surgical ablation.

After 2014, research emphasis shifted to keywords including “reticular pseudodrusen,” “geographic atrophy (GA),” and “aflibercept.” Reticular pseudodrusen was confirmed as a key precursor lesion of MNV3, with its presence increasing the risk of progression to geographic atrophy (34, 35). GA, a common post-treatment complication, became a research hotspot because of its pathological connection to abnormal vascular proliferation, driving efforts to optimize long-term disease management (9). Aflibercept gained prominence for its potent and sustained VEGF inhibition (36). Collectively, this shift highlighted priorities in precursor lesions, therapy-pathology interactions and treatment refinement. The subsequent emergence of “type 3 macular neovascularization” in 2021 (burst strength: 13.08) marked the subfield’s maturation.

### 3.6 Analysis of co-cited references

#### 3.6.1 Top co-cited references

Table 5 displays the top 10 co-cited references. Four focus on the definition and spectrum expansion of MNV3 (22, 32, 37, 38), three address the clinical risks and early identification of MNV3 (39–41), and another three concern diagnosis, treatment and imaging applications of MNV3 (42–44). The most highly cited reference systematically elaborated on the clinical characteristics of RAP in AMD, laying the foundation for the definition and classification of this lesion (32). The second-ranked study expanded the disease spectrum of type 3 neovascularization and clarified its association with MNV3 (37). It is worth noting that relevant studies have emphasized that the unaffected fellow eyes of patients with monocular MNV3 carry a high risk of neovascular lesions and require close monitoring (39). Meanwhile, a study enrolling 104 cases preliminarily explored the treatment regimens and efficacy in MNV3, providing early practical references for clinical intervention (42). The application of spectral-domain optical coherence tomography (SD-OCT) technology has further clearly revealed the evolution law of MNV3, its pathological association with pigment epithelial detachment (PED), and treatment response, promoting the precise diagnosis and treatment process of such lesions (44).

**TABLE 5:** Top 10 co-cited references.

Rank	Citation counts	Author	Reference title	Journal	Year
1	210	Yannuzzi LA	Retinal Angiomatous Proliferation in Age-related Macular Degeneration	Retina-J Ret Vit Dis	2001
2	128	Freund KB	Type 3 Neovascularization: The Expanded Spectrum of Retinal Angiomatous Proliferation	Retina-J Ret Vit Dis	2008
3	74	Gross NE	Nature and Risk of Neovascularization in the Fellow Eye of Patients With Unilateral Retinal Angiomatous Proliferation	Retina-J Ret Vit Dis	2005
4	74	Slakter JS	Retinal Choroidal Anastomoses and Occult Choroidal Neovascularization in Age-Related Macular Degeneration	Ophthalmology	2000
5	71	Bottoni F	Treatment of Retinal Angiomatous Proliferation in Age-Related Macular Degeneration: A Series of 104 Cases of Retinal Angiomatous Proliferation	Arch Ophthalmol Chic	2005
6	70	Gass JDM	Focal Inner Retinal Hemorrhages in Patients With Drusen: An Early Sign of Occult Choroidal Neovascularization and Chorioretinal Anastomosis	Retina-J Ret Vit Dis	2003
7	69	Hartnett ME	Deep Retinal Vascular Anomalous Complexes in Advanced Age-Related Macular Degeneration	Ophthalmology	1996
8	65	Yannuzzi LA	Review of Retinal Angiomatous Proliferation or Type 3 Neovascularization	Retina-J Ret Vit Dis	2008
9	60	Kuhn D	Imaging of Chorioretinal Anastomoses in Vascularized Retinal Pigment Epithelium Detachments	Arch Ophthalmol Chic	1995
10	58	Nagiel A	Type 3 Neovascularization: Evolution, Association With Pigment Epithelial Detachment, and Treatment Response as Revealed by Spectral Domain Optical Coherence Tomography	Retina-J Ret Vit Dis	2015

#### 3.6.2 Eleven clusters of the co-citation network and cluster dependencies

**FIGURE 5:**
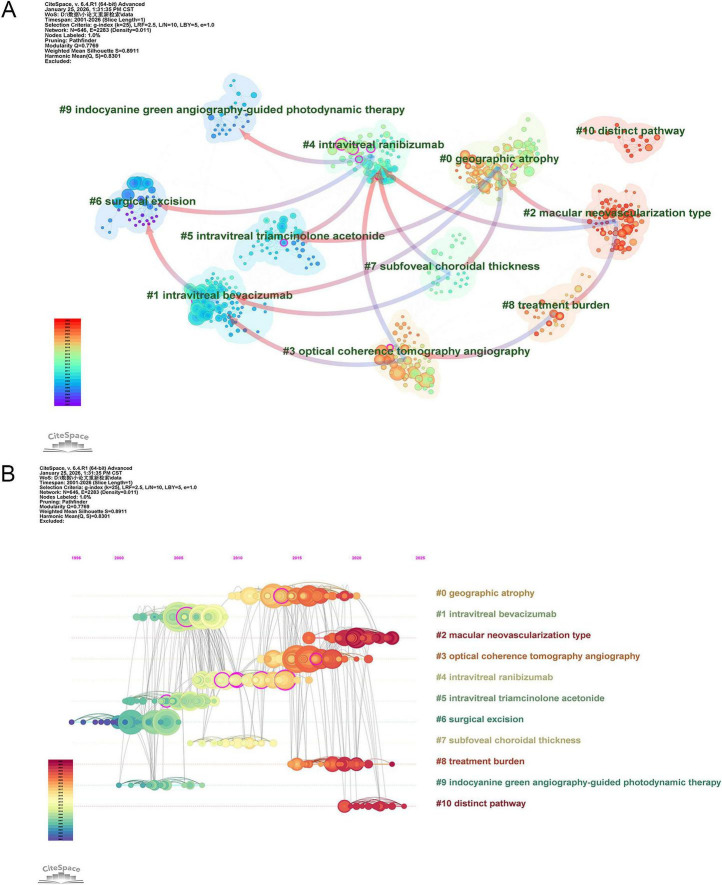
The main co-citation clusters. **(A)** Visualization of the top 11 co-cited clusters. Terms from the title field of the citing papers within each cluster are adopted as the definition of that cluster. Arrows point from the citing clusters to the cited clusters. **(B)** Timeline view of these listed clusters of the co-cited references.

The co-citation network was partitioned using the Log-Likelihood Ratio (LLR) algorithm, yielding distinct communities with significant modularity (*Q* = 0.777) and silhouette (*S* = 0.891). As shown in Figure 5A, the top 11 major themes are: #0 geographic atrophy, #1 intravitreal bevacizumab, #2 macular neovascularization type, #3 optical coherence tomography angiography, #4 intravitreal ranibizumab, #5 intravitreal triamcinolone acetonide, #6 surgical excision, #7 subfoveal choroidal thickness, #8 treatment burden, #9 indocyanine green angiography-guided photodynamic therapy and #10 distinct way. Three clusters (#0, #2, #10) examine MNV3 pathology and risk factors, two (#3, #7) address imaging-based diagnosis and evaluation, five (#1, #4, #5, #6, #9) revolve around treatment strategies, and one (#8) pertains to clinical management. Arrows between clusters indicate citation relationships, pointing from citing to cited groups. Cited collections generally represent foundational work, while those positioned to the right often correspond to the latest developments.

#### 3.6.3 Timeline map of clusters

The timeline view (Figure 5B) traces the emergence and evolution of research clusters over time. In the early stage, surgical excision (#6), a traumatic procedure, was the primary clinical intervention, reflecting the limited understanding of MNV3 pathogenesis at that time. As the role of VEGF as a core driver of neovascularization became established, research focus shifted toward targeted therapies. Between 2005 and 2015, anti-VEGF agents (#1, #4), triamcinolone acetonide (#5), and photodynamic therapy (#9) became research hotspots, revolutionizing the clinical treatment paradigm for MNV3.

However, long-term clinical practice has exposed limitations of these therapies. Although anti-VEGF treatments effectively inhibit neovascularization, they may accelerate the progression of geographic atrophy (#0), a key issue affecting long-term prognosis. In addition, frequent drug injections increase treatment burden (#8), prompting studies on the economic and quality-of-life impacts of long-term management. Concurrently, advances in imaging techniques such as optical coherence tomography angiography (#3) have enhanced the ability to accurately assess MNV3 lesion dynamics, providing more refined tools for mechanistic research and efficacy evaluation.

#### 3.6.4 Details of cluster #0 (geographic atrophy), #2 (macular neovascularization type) and #10 (distinct way)

These three clusters are related to the pathology of MNV3. As a unique subtype of nAMD, MNV3 is characterized by retinal-choroidal anastomosis (RCA) (45). The lesions mostly avoid the macular fovea and are mainly distributed in the temporal side of the fovea (46, 47). A special subtype (cMNV3), originating from the cilioretinal artery, represents 12% of cases and is often accompanied by exudative macular lesions and intraretinal hemorrhage (48). Histologically, MNV3 originates from the deep retinal capillary plexus (49), and its occurrence and development are closely related to the increase of pro-inflammatory cytokines (50). In addition, perfusion studies have shown that compared with other subtypes, patients with MNV3 have more significant choroidal damage. Even in patients with unilateral MNV3, the choroidal dysfunction in the contralateral unaffected fellow eye is more severe than that in the contralateral eye of patients with MNV1/2 (51).

Geographic atrophy (#0)-relevant literature has confirmed that MNV3 is an independent baseline risk factor for the development of GA, along with subfoveal choroidal thinning, RPD and GA in the fellow eye (52–54). In 41 treatment-naive eyes with MNV3, MA incidence reached 92.7% after 12 months of anti-VEGF treatment (55). Some treatment-naive eyes already have atrophy at baseline, and new or expanding GA is prone to occur during follow-up (54–57). The expansion of the RPE atrophy area in MNV3 eyes is negatively correlated with central choroidal thickness and positively correlated with the number of anti-VEGF injections. Ranibizumab treatment and monthly dosing regimens increase the risk of GA development (52, 58). Additional studies have indicated that RPD serves as a precursor lesion and a risk factor for bilateral MNV3 (59, 60). Its prevalence is markedly higher in MNV3 than in typical nAMD, and is associated with reduced choroidal thickness (34, 35). Focal RPE elevations found in the fellow eye have been identified as potential precursors to MNV3, which progressively evolve into the focal atrophy of RPE and photoreceptor (61).

Regarding lesion evolution, the MNV3 complex does not completely regress after anti-VEGF treatment, and changes in vascular branches correlate with treatment response (62). MNV3-related MA arises via two distinct pathways: one driven by neovascularization and exudation of MNV3, the other by basic AMD lesions independent of the neovascularization (55). The prevalence of subretinal fibrosis (SRFi) in nAMD eyes with MNV3 increases with treatment duration, and poor baseline visual acuity is an independent risk factor (63).

#### 3.6.5 Details of cluster #3 (optical coherence tomography angiography) and #7 (subfoveal choroidal thickness)

Optical coherence tomography angiography (OCTA), as a non-invasive imaging technique, can clearly display the retinal microvascular structure and is of great value in the diagnosis of MNV3 and the monitoring of treatment responses (64, 65). This technique can effectively identify small intraretinal neovascular complexes connected to the deep retinal capillary plexus (66), and distinguish vascular from non-vascular lesions through linear high-flow characteristics (65). Follow-up observations have shown that the choroidal capillary blood flow signals associated with abnormal lesions in the outer retina can disappear or evolve into subretinal pigment epithelial neovascularization after anti-VEGF treatment (67). In addition, compared with two-dimensional OCTA, the rotating three-dimensional OCTA helps to further improve the detection rate of lesions (68).

Meanwhile, multiple comparative studies have shown that the choroidal thickness in patients with MNV3 is significantly thinner than that in patients with typical nAMD and normal populations (69, 70), and there is no significant difference between stages two and three, suggesting that a thin choroid may be an intrinsic feature of MNV3 (70). After combined treatment, the choroidal thickness of the affected eye can continuously decrease, and this is not related to the recurrence of the lesion (71).

#### 3.6.6 Details of cluster #1 (intravitreal bevacizumab), #4 (intravitreal ranibizumab), #5 (intravitreal triamcinolone acetonide), #6 (surgical excision) and #9 (indocyanine green angiography-guided photodynamic therapy)

These five clusters center on therapeutic strategies for MNV3. Surgical treatment was one of the earliest approaches. For stage II MNV3, surgical ablation demonstrated short-term efficacy (72). However, subsequent investigations revealed a 100% recurrence rate within 2–13 months postoperatively (73). This high recurrence persists even with combined PDT, indicating limited utility of surgical approaches for stage II/III MNV3 (74). Laser photocoagulation, including micro-pulse, focal, and argon laser techniques, has shown favorable outcomes for early-stage lesions not involving the fovea (75–77).

Clusters #5 (intravitreal triamcinolone acetonide) and #9 (indocyanine green angiography-guided photodynamic therapy) mainly include the transitional stage of MNV3 treatment research. These two methods are often used as adjuvant approaches in combined therapy. Triamcinolone exerts anti-inflammatory effects and alleviates macular edema induced by MNV3. PDT combined with intravitreal triamcinolone injections can lead to a reduction or elimination of edema, rapid regression of neovascularization, and stabilization or improvement of visual acuity (78, 79).

Clusters #1 (intravitreal bevacizumab) and #4 (intravitreal ranibizumab) are the core clusters in current MNV3 treatment research, centering primarily on the efficacy validation and protocol optimization for anti-VEGF agents. The standard protocol involves an initial series of three monthly loading injections, followed by on-demand treatments (80, 81). Anti-VEGF monotherapy yields favorable outcomes in stage I/II lesions. For patients with a poor response to monotherapy or severe exudation, combining anti-VEGF therapy with PDT has been shown to significantly augment both functional and anatomical outcomes while reducing the frequency of injections (82). However, this regimen increases the risk of GA in patients with baseline RPD or a greater lesion diameter (GLD) (83). Notably, visual improvement is limited in patients with stage III MNV3 irrespective of the regimen, necessitating repeated injections (84, 85). In contrast, triamcinolone–PDT combination achieves superior functional and anatomical outcomes compared with ranibizumab monotherapy or ranibizumab–PDT combination therapy, albeit with a higher risk of GA and other complications (86).

#### 3.6.7 Details of cluster #8 (treatment burden)

Compared with patients with MNV1/2 and PCV, patients with MNV3 have a significantly higher annual frequency of anti-VEGF injections and visit rates, as well as a higher proportion of bilateral involvement, which together constitute a heavy objective treatment burden (87). There is a trade-off between efficacy and injection frequency among different treatment regimens, such as PRN and TAE (23).

In terms of regional and population characteristics, MNV3 is more common in Western Europe and the Mediterranean region. These regions have longer life expectancy and more elderly patients. The large elderly population has relatively poor tolerance to long-term treatment, which further increases the overall local treatment burden (88).

In addition, patients with MNV3 also face significant psychological and social challenges: nearly half of the patients experience anxiety or depression, which is closely related to the decline in health-related quality of life. At the same time, visual impairment may increase patients’ dependence on their families and lead to difficulties in adapting to social roles (89, 90).

Geographical barriers and insufficient social support further exacerbate the overall burden. Long-distance visits and limited insurance coverage further aggravate the overall burden (90).

## 4 Discussion

### 4.1 Leading countries, top authors, top co-cited authors, and leading journals

In terms of national distribution, the United States dominates MNV3 research with 3,289 citations and the highest centrality (Table 1). Moreover, more than half of the highly co-cited authors are from the United States (Table 2), which may be attributed to its stable funding support and mature scientific research system. The high centrality of China, Italy, and Singapore highlights their important roles in the collaboration network. Although the United Kingdom has a relatively low publication volume, it demonstrates outstanding citation impact, reflecting strong academic influence.

Regarding authors, there is little overlap between highly productive authors and highly co-cited authors, suggesting a discrepancy between publication quantity and global co-citation influence. Kim JW is the most prolific author, and he has maintained close collaboration with Kim CG, Lee DW, and Chang YS, all affiliated with Konyang University in South Korea. Their early research concentrated on diagnostic methods for MNV3 (69, 91), and validation of anti-VEGF efficacy (57, 92). In the middle stage, he shifted to optimizing therapeutic regimens (23, 93), and investigating MNV3-related complications (94, 95). Later, he integrated cutting-edge technologies to study the dynamic evolution and risk prediction of MNV3 (96–99). In contrast, highly co-cited authors are predominantly American scholars. Hartnett ME first reported the disease, while the teams of Yannuzzi LA, Freund KB, Spaide RF, and Gross NE laid the foundation for the classification and nomenclature of MNV3. They formally proposed and defined MNV3 as an independent subtype of AMD, establishing the core concept and expanded spectrum of the disease (32, 37). They developed a staging system for MNV3 centered on SD-OCT (100). They were the first to apply the TAE regimen to anti-VEGF therapy for MNV3 (101). Furthermore, they systematically elucidated the natural course, bilateral onset risk, and prognostic patterns of MNV3, offering core evidence-based support for long-term patient management (39).

In terms of journals, research outputs are highly concentrated in *IOVS* and *Retina*, with *Retina* also being the most highly cited journal, reflecting its status as a core platform. There is substantial overlap between highly productive journals and highly co-cited journals, confirming that these platforms serve as key vehicles for disseminating high-impact MNV3 research.

### 4.2 Development trend in MNV3

Co-occurrence and burst analyses of keywords could provide insights into research status, hotspots of different directions, and the evolution of frontiers in this field. Co-citation analysis can uncover the core knowledge foundation and classic theoretical frameworks. In this study, the research directions identified by the co-occurrence network and keyword bursts were generally consistent (Figures 3A, 4) and could be grouped into three major fields: (1) pathophysiology, (2) imaging, and (3) therapy. In combination with co-citation analysis, we further analyze the core foundations and frontiers of each field.

#### 4.2.1 Pathophysiology domain

Phenotypically, the understanding of MNV3 pathophysiology has evolved through years of research. Evidence of this evolution can be captured from keyword bursts and highly co-cited literature (Figure 4 and Table 5). The disease now known as MNV3 was first described as “deep-retinal vascular anomalous complexes associated with PEDs” in 1992 (21, 22). In 1995, Kuhn et al. proposed the “chorioretinal anastomosis” hypothesis, suggesting that neovascularization originates from the choroid. Histopathological and angiographic investigations subsequently revealed that these lesions exhibited angiomatous proliferation and originated from the inner retinal vascular network rather than the choroid. Accordingly, Yannuzzi et al. formally designated the entity as “RAP” and proposed a three-stage classification system based on the origin and progression of the aberrant vessels (32). This represents a major shift in the understanding of MNV3. Expanding on this, Freund et al. analyzed neovascular characteristics in a larger cohort and introduced a more descriptive term, “type 3 neovascularization” (37). With advancements in imaging, the staging system was later refined using SD-OCT to focus on the correlation between evolving retinal punctate hyperreflective lesions and retinal layer disruption (100). Integrating these pathological and imaging insights, the 2020 international clinical consensus formally unified the entity as “MNV3” (5).

Mechanistically, MNV3 is generally considered to result from interactions between environmental and genetic factors. Relevant keywords can be identified from the keyword co-occurrence network (Figure 3).

Environmentally, the core triggers are chronic hypoxia and oxidative stress, mainly mediated through the von Hippel-Lindau (VHL)-hypoxia-inducible factor (HIF) signaling pathway. Chronic hypoxia in the paracentral outer retina, driven by Bruch’s membrane thickening, reduced choroidal blood flow, and drusen accumulation, inhibits HIF-1α degradation and leads to its sustained accumulation, thereby activating pro-angiogenic genes (102, 103). VHL dysfunction further exacerbates HIF-1α accumulation. Abnormal activation of the mammalian target of rapamycin (mTOR) pathway amplifies the pro-angiogenic effect of HIF-1α (104). Conversely, the retinoblastoma protein (Rb) antagonizes this effect by directly binding and inhibiting the transcriptional activity of HIF-1α, jointly establishing a regulatory balance with the abnormally activated mTOR (104, 105).

Genetically, MNV3 is associated with multiple genetic variants, primarily involving two core pathways: extracellular matrix homeostasis (e.g., age-related maculopathy susceptibility 2, *ARMS2*) and the complement system (e.g., complement factor H, *CFH*). Additional susceptibility variants are also related to lipid metabolism (e.g., very low-density lipoprotein receptor, *VLDLR*), vascular development (e.g., *VEGF*), and inflammatory processes.

The *ARMS2* A69S genotype is strongly associated with MNV3 and elevates the risk of RPD (34, 106). In contrast, the *CFH* I62V variant has a weaker effect, and the *CFH* Y402H variant shows no or low statistical correlation with RAP (34, 107). Complement system dysregulation causes damage to the RPE cell membrane and promotes the release of inflammatory cytokines and oxidative stress products, further exacerbating RPE metabolic dysfunction (108). The *CFH* I62V mutation also disrupts zonula occludens-1 (ZO-1) expression, compromising the blood-retinal barrier and causing outer retinal hypoxia (109). Deletion of the *VLDLR* gene activates inflammation, which, together with accumulated HIF-1α, significantly enhances the expression of angiogenic factors and promotes neovascularization (110, 111).

#### 4.2.2 Imaging domain

Keyword burst analysis (Figure 4) reveals a clear, phased evolutionary trajectory of imaging technologies for MNV3. Between 2001 and 2013, indocyanine green angiography (ICGA) emerged as the primary burst keyword for imaging in MNV3. Its high sensitivity for vascular anastomoses and lesion “hot spots” effectively overcame key limitations of fluorescein angiography (FA) in visualizing MNV3, providing a critical basis for its early classification and definition (33). From 2015 to 2024, OCTA arose as the new central burst keyword, representing a paradigm shift toward contrast-free, non-invasive 3D imaging of the deep retinal microvasculature, which has since become the mainstream modality in research and clinical practice (65, 112).

In the keyword co-occurrence network (Figure 3), we can observe imaging-related terms such as OCT, SD-OCT, Swept-source OCT (SS-OCT), and Enhanced Depth Imaging OCT (EDI-OCT). These OCT-derived techniques further establish an integrated evaluation system for the structural and blood-flow characteristics of MNV3. SD-OCT exhibits high sensitivity in identifying hyperreflective foci of neovascularization and RPE rupture (100). SS-OCT supports the identification of very early and subtle MNV3 lesions, particularly in eyes with opaque media (113, 114). EDI-OCT permits in-depth assessment of choroidal thickness in MNV3, and phase-resolved Doppler OCT further elevates imaging capabilities in deeper tissues (69, 115). En face OCT complements cross-sectional imaging to clarify lesion morphology and its spatial relationship to the fovea (116). Building on the aforementioned imaging modalities, multimodal imaging integrates the strengths of various modalities, providing key evidence for exploring the pathogenesis of MNV3 (103).

In recent years, the application of AI algorithms, such as deep learning, has led to significant breakthroughs in diagnostic imaging. A multi-input deep learning model rooted in a refined Visual Geometry Group (VGG) network predicted the 24-month neovascularization risk in the contralateral eye with accuracy exceeding that of junior ophthalmologists (98). Another optimized VGG-19 model could reliably differentiate MNV3 from polypoidal choroidal vasculopathy (PCV), with efficacy comparable to that of 8 ophthalmologists (including 2 senior experts) (12).

#### 4.2.3 Therapy domain

The treatment-related development trends revealed by keyword burst analysis and reference co-citation clustering are highly consistent (Figures 4, 5B). Early research focused on invasive interventions, which gradually declined due to severe trauma and a high postoperative recurrence rate. Subsequently, triamcinolone acetonide and PDT became research hotspots. Relying on the precise lesion-targeting advantage of ICGA, the focus of treatment formally shifted to minimally invasive local interventions and pharmacotherapy.

Currently, research on anti-VEGF therapy is dominant, with its scope gradually expanding from drug selection to the effects of baseline characteristics on treatment outcomes and personalized treatment regimens, among other areas. Literature indicates that in MNV3 patients with thin choroids, RPD, contralateral GA, or long-term visual protection requirements, ranibizumab is associated with a lower risk of GA than aflibercept (83, 92). In terms of dosing strategies, studies have explored differentiated regimens based on diverse baseline and risk characteristics. A PRN approach is preferentially adopted in patients with retinitis pigmentosa (RP) or large PED to avoid risks associated with frequent injections (94, 117). PRN is also the main strategy for clinically stable patients, whereas TAE therapy is preferred for high-risk recurrent cases, which shows lower rates of RPE tears and sub macular hemorrhage than PRN but require more injections (93). For MNV3 cases complicated with severe RPE damage, subretinal surgery combined with autologous RPE transplantation has been reported as a feasible rescue therapy (118). For patients with high treatment burden, biomarker-based individualized strategies have been widely investigated, with an initial PRN followed by an active extended regimen upon recurrence (23, 119–121). Moreover, follow-up may be prolonged in recurrence-free cases, and injection frequency reduced in patients with RPE atrophy (87, 122, 123).

It is clear that anti-VEGF-based therapies face certain limitations. Fortunately, novel approaches such as HIF-1α inhibitors, VEGF inhibitors combined with Angiopoietin-2 (ANG2) antagonists, or interventions targeting the PI3K pathway are gradually advancing, making effective treatment of advanced MNV3 a possibility (10, 11, 104, 105).

### 4.3 Limitations of our study

This study has several inherent limitations. First, PubMed lacks standardized and structured citation data, rendering it unsuitable for co-citation analysis. Therefore, this study employed the WoSCC to perform co-citation analysis, serving as a supplementary validation to the keyword co-occurrence analysis. Although the overall research themes and developmental trends identified by the two methods were generally consistent, discrepancies in indexing coverage between PubMed and WoSCC may still introduce a certain degree of publication bias. Second, the exclusive use of CiteSpace and VOSviewer for bibliometric analysis may introduce inherent analytical limitations. Specifically, the keyword co-occurrence clusters derived from VOSviewer are based on modularity optimization rather than semantic similarity. As such, the labeling of clusters represents only a broad thematic summary of research priorities and does not constitute a strict, semantically precise classification. Finally, the evolving complexity of MNV3 research makes a fully comprehensive bibliometric analysis challenging. Despite these limitations, we have striven to detail the research hotspots and trends within this subject as thoroughly as possible.

## 5 Conclusion

Bibliometric analysis indicates that current research on MNV3 still holds significant potential for development. The United States leads in both the number of publications and citation counts. *IOVS* and *Retina* rank first in published papers and citations, respectively. In recent years, MNV3 research has predominantly concentrated on pathophysiology, imaging techniques and therapeutic approaches. Future research directions entail developing animal models to elucidate its pathogenesis for targeted drug discovery, and leveraging advanced imaging technologies and computational science to enhance clinical diagnostic accuracy.

## Data Availability

The original contributions presented in the study are included in the article/supplementary material, further inquiries can be directed to the corresponding author.
